# Supplementary material for: Zika virus infection induces host inflammatory responses by facilitating NLRP3 inflammasome assembly and interleukin-1β secretion
Source: Nat Commun. 2018 Jan 9;9:106. doi: 10.1038/s41467-017-02645-3 (PMC5760693; doi:10.1038/s41467-017-02645-3)
Supplement: Supplementary file 1 — Supplementary Information [file 41467_2017_2645_MOESM1_ESM.pdf]

Supplementary Figures and Legends

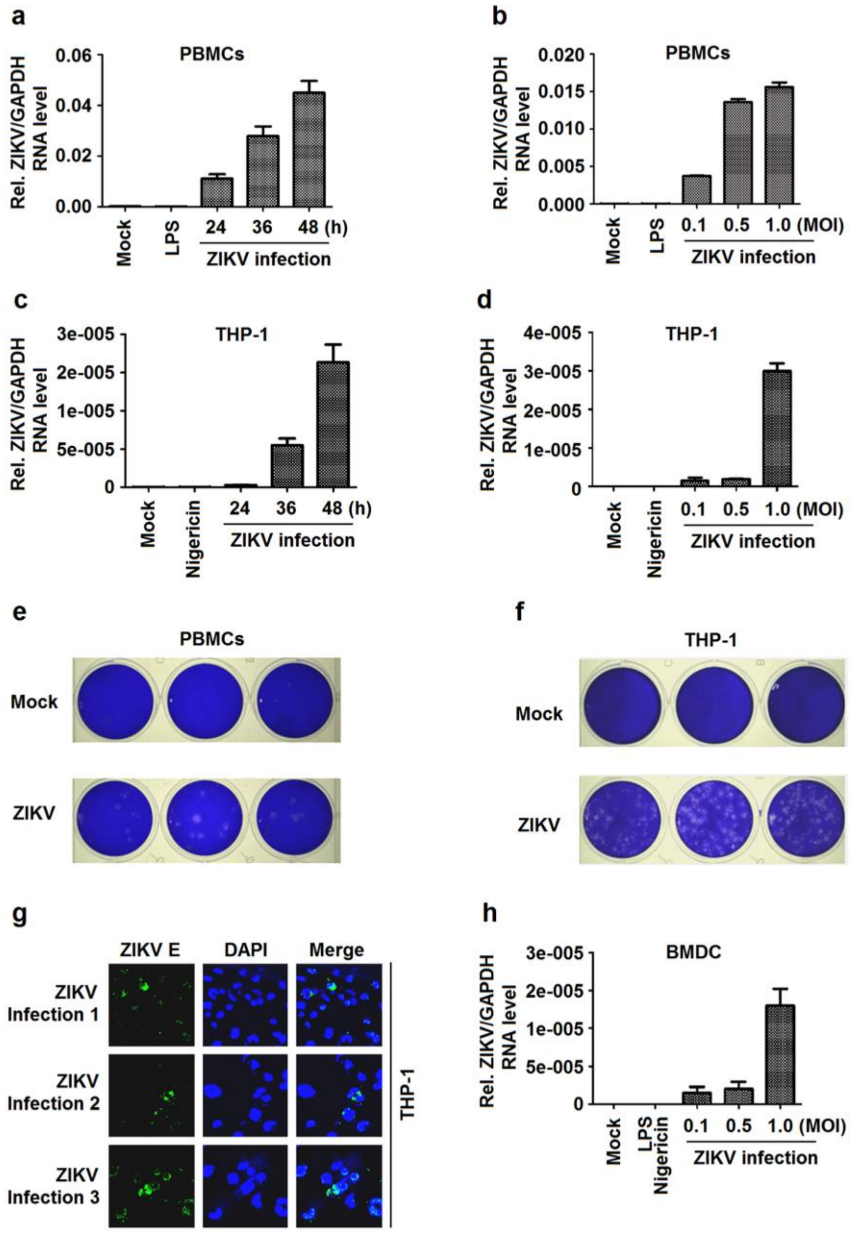

Supplementary Fig. 1. Determination of ZIKV RNA in infected cells. (**a**–**d**) Human PBMCs isolated from healthy individuals were stimulated with LPS (1 μg per ml) for 6 h (a and b) or 2 μM Nigericin for 2 h (c and d), infected with ZIKV at MOI = 1 for 24, 36, or 48 h (a and c) or infected with ZIKV for 48 h at MOI = 0.1, 0.5, or 1 (b and d). The levels of ZIKV mRNA and *GAPDH* mRNA were quantified by RT-PCR. (**e**–**g**) Human PBMCs isolated from healthy individuals (e) or TPA-differentiated THP-1 macrophages (f and g) were infected with or without ZIKV at MOI=1 for 24, 36, and 48 h. The production of ZIKV in the PBMCs cell supernatant was analysis by the plaque assay (e and f). The supernatant was diluted in 10-fold. The infection of ZIKV in THP-1 macrophages was detected by confocal microscopy (g). Sub-cellular localizations of ZIKV E protein (green) and the nucleus

marker DAPI (blue) were examined by confocal microscopy. (**h**) Mouse BMDCs were infected by ZIKV for 48 h at MOI=0.1, 0.5n and 1 or stimulated by LPS (1 μg per ml) for 6 h and 2 μM Nigericin for 30 min. The levels of ZIKV mRNA and *GAPDH* mRNA were quantified by RT-PCR. The number of replicates is three (a, b, c, d, and h). Data shown are means ± s.e.m.

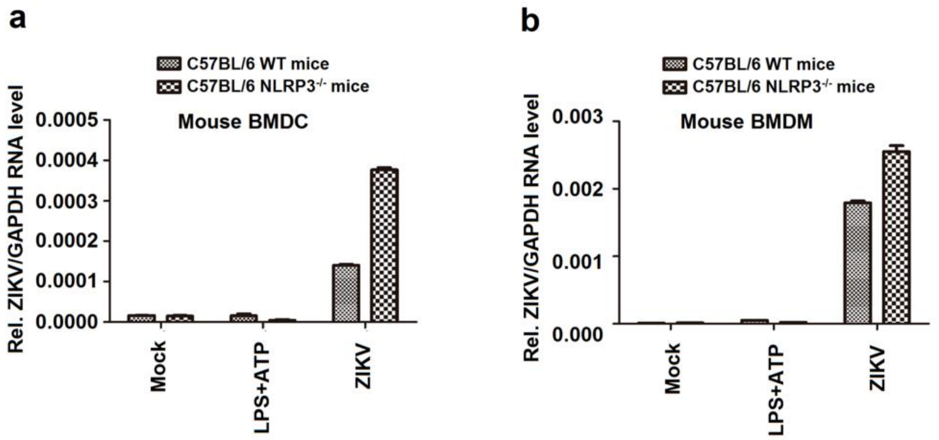

Supplementary Fig. 2. Analyses of ZIKV RNA in infected mice. (**a** and **b**) Mouse BMDCs were prepared from bone marrow cells of treated C57BL/6 mice and *NLRP3*$^{-/-}$ mice (a). Mouse BMDMs were then prepared from bone marrow cells of treated C57BL/6 mice and *NLRP3*$^{-/-}$ mice (b). The cells form C57BL/6 WT mice and NLRP3$^{-/-}$ mice were stimulated with LPS (1 µg per ml) for 6 h and 5 mM ATP for 30 min or infected with ZIKV for 24 h at MOI=1. The levels for ZIKV RNA and *GAPDH* RNA were quantified by real-time PCR. The number of replicates is three. Data shown are means ± s.e.m.

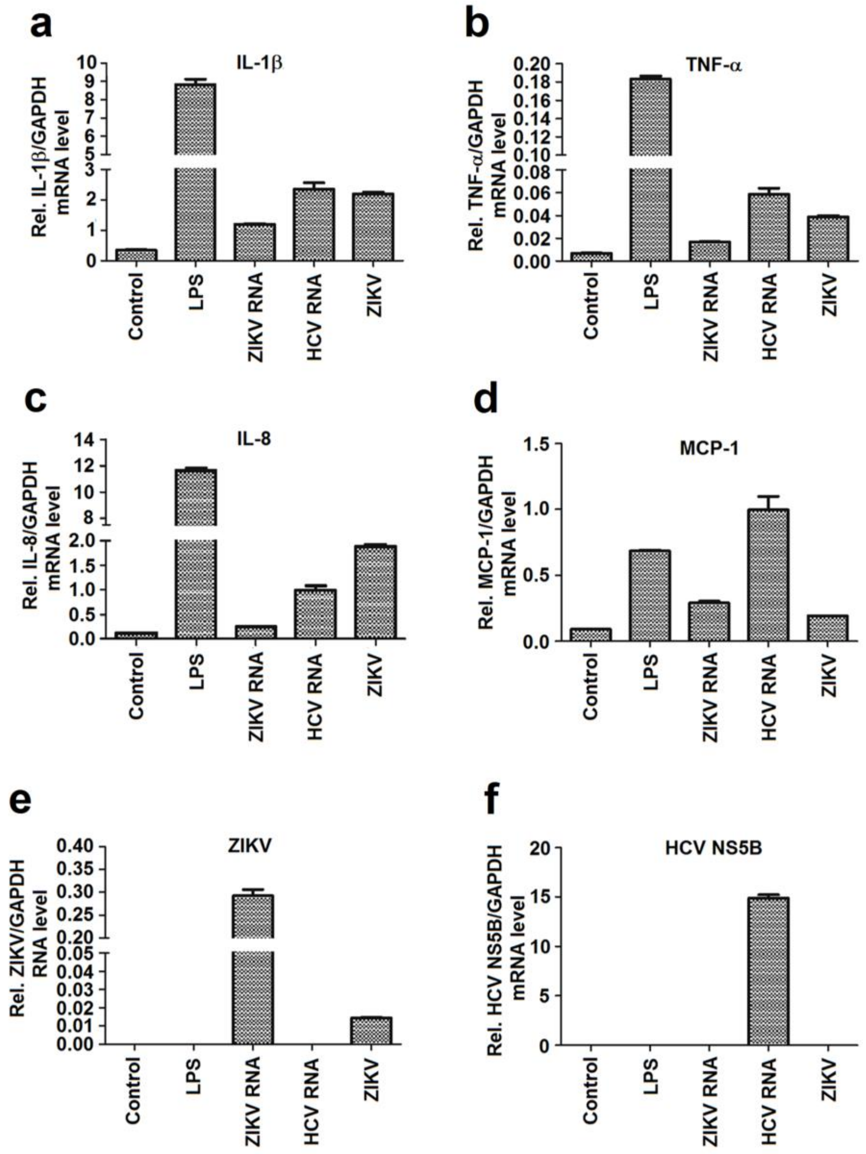

Supplementary Fig. 3. The effect of ZIKV genomic RNA on the expression of NF-κB-targeted genes. (**a**–**f**) TPA-differentiated THP-1 cells were stimulated for 6 h with Lipo, LPS, ZIKV genomic RNA (5 µg per ml), HCV genomic RNA (5 µg per ml), or infected with ZIKV for 24 h. The levels for *IL-1β* mRNA (a), *TNF-α* mRNA (b), *IL-8* mRNA (c), *MCP-1* mRNA (d), ZIKV mRNA (e) and HCV *NS5B* mRNA (f) were quantified by qRT-PCR. The number of replicates is three. Data shown are means ± s.e.m.

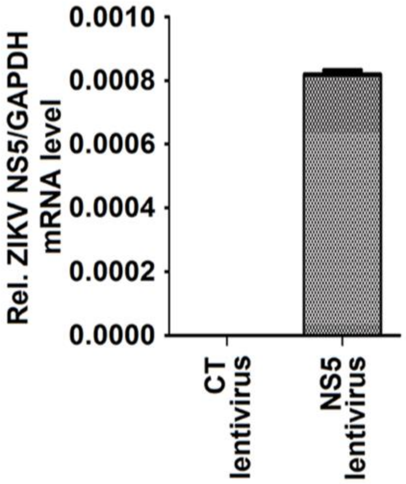

Supplementary Fig. 4. Determination of the efficiency of NS5 lentivirus. TPA-differentiated THP-1 macrophages were infected with control lentivirus (CT-lentivirus) or NS5-expresing lentivirus (NS5-lentivirus). The levels of *NS5* RNA and *GAPDH* mRNA expressed in the stable cells were determined by qRT-PCR. The number of replicates is three. Data shown are means ± s.e.m.

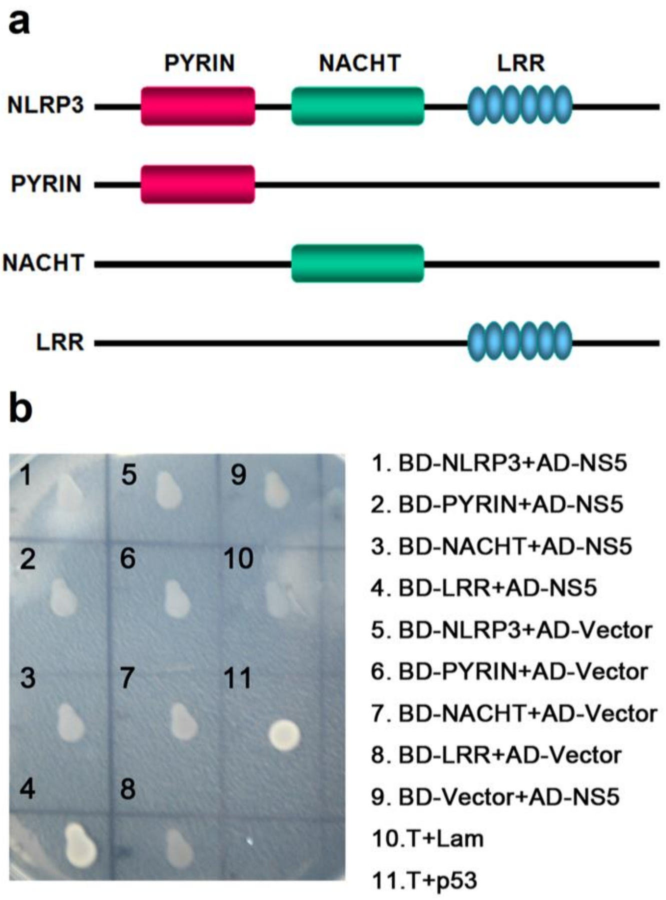

Supplementary Fig. 5. Yeast two-hybrid analyses of the interaction between NS5 and NLRP3 or its domains. (**a**) Diagrams of the structures of NLRP3 protein, NLRP3 PYRIN domain, NLRP3 NACHT domain and NLRP3 LRR domain. The numbers indicated the locations of amino acid sequences. (**b**) Yeast strain AH109 cells were transformed with the combination of BD and AD plasmid, as indicated. Transformed yeast cells were first grown on the SD-minus Trp/Leu plates for three days. The colony of yeast was then streaked on SD-minus Trp/Leu/Ade/His plates (QDO) for two days. BD-p53 and AD-T was used as a positive control and BD-lam and AD-T as a negative control.

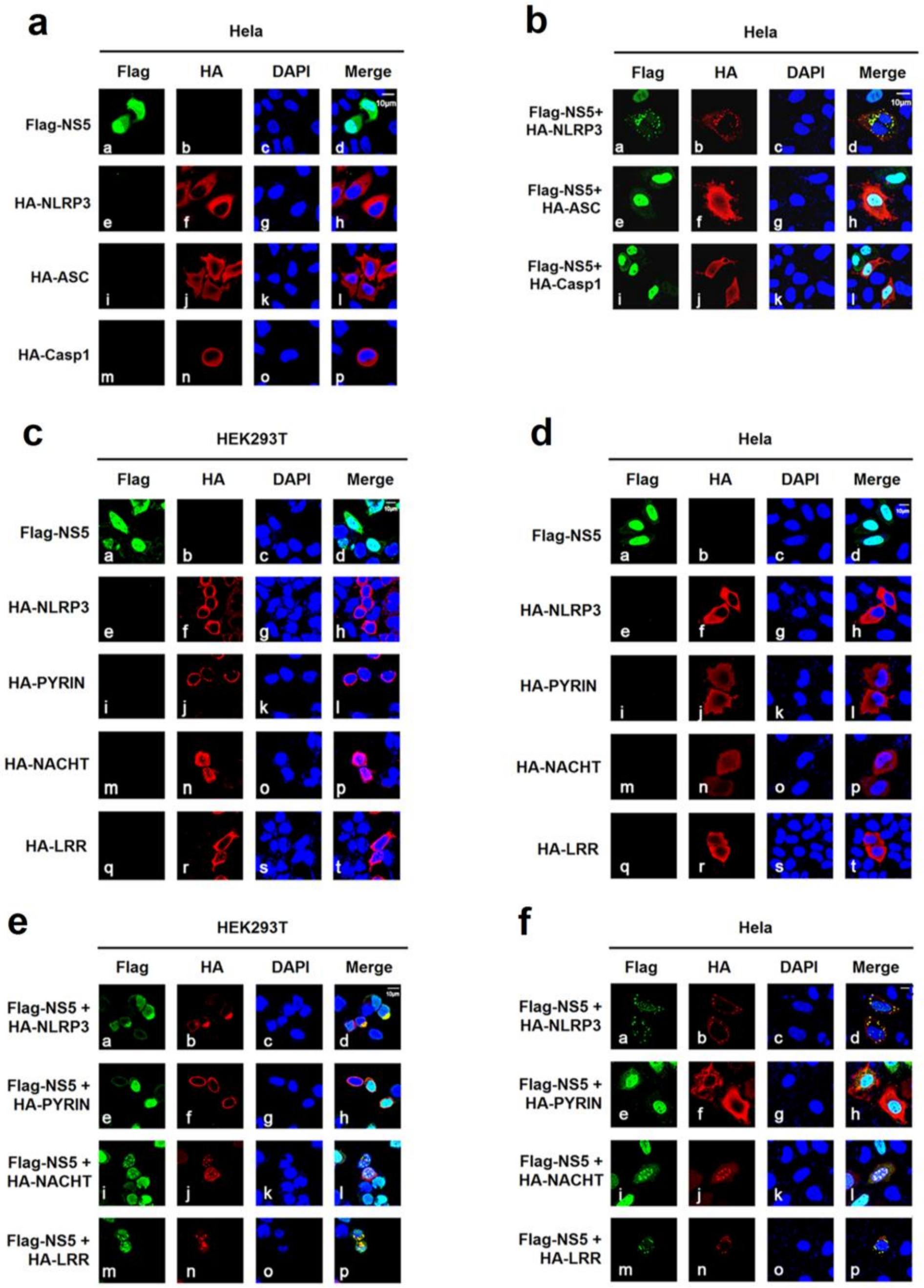

Supplementary Fig. 6. The co-localization of NS5 with NLRP3 and NACHT and LRR domains. (**a** and **b**) Hela cells were transfected with pFlag-NS5, pHA-NLRP3, pHA-ASC or pHA-Casp-1 (a) or transfected with pFlag-NS5 and pHA-NLRP3, pHA-ASC or pHA-Casp-1 (b). (**c** and **d**) HEK293T cells (c) and Hela cells (d) were transfected with pFlag-NS5, pHA-NLRP3, pHA-PYRIN, pHA-NACHT or pHA-LRR. (**e** and **f**) HEK293T cells (e) and Hela cells (f) or co-transfected with pFlag-NS5 and pHA-NLRP3, pHA-PYRIN, pHA-NACHT or pHA-LRR. (**a**–**f**) The sub-cellular localizations of Flag-NS5 (green), HA-protein (red), nucleus marker DAPI (blue) and merge were examined under confocal microscopy. Scale bar is 10 μm.

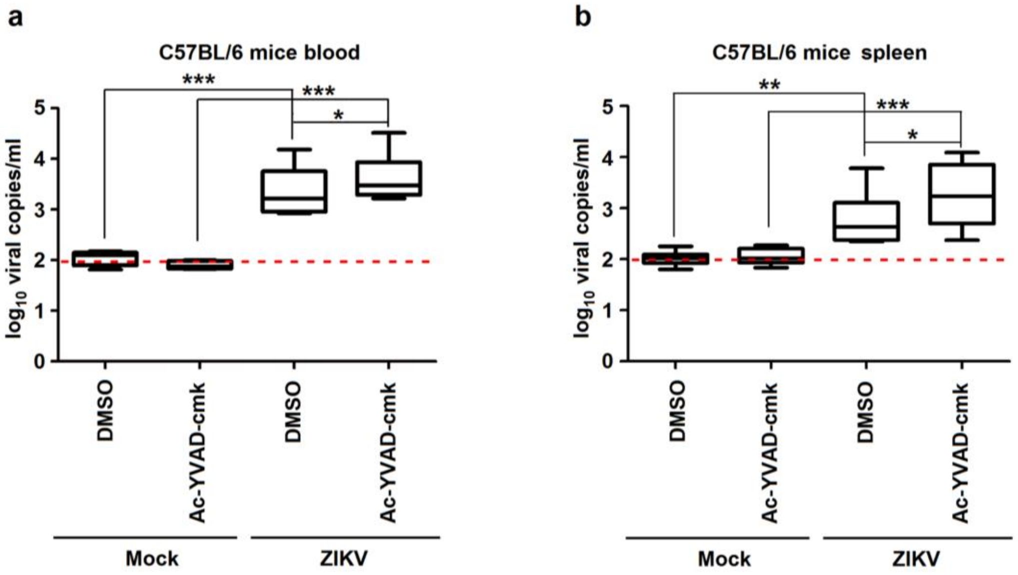

Supplementary Fig. 7. The detection of ZIKV infection in mice spleen. (**a** and **b**) C57BL/6 WT mice (5 weeks old, male) were pretreated with DMSO (n = 6) or Ac-YVAD-cmk (8 mg per kg) (n =6) by intraperitoneal injection for 30 mine, and then infected with ZIKV (5×10^5 PFU) (n = 6) or treated with PBS (n = 6) for 2 days. Viral titers in mice blood were determined by RT-PCR (a). Viral titers in mice spleen were determined by RT-PCR (b). Data shown are Whiskers: Min to Max; *P < 0.05, **P < 0.01,***P < 0.0001 (one-way ANOVA with Tukey’s post-hoc test).

Uncropped Figure 2B

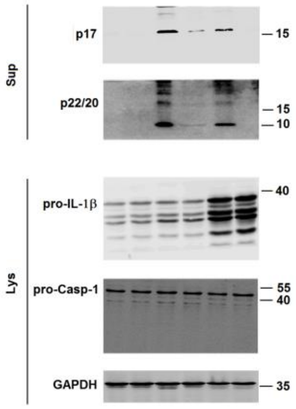

Uncropped Figure 2D

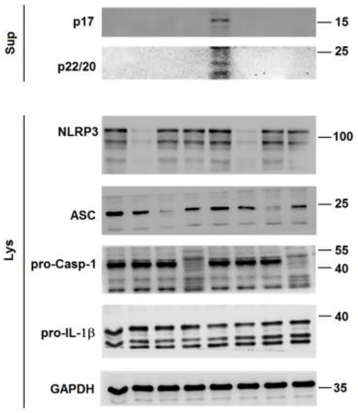

Uncropped Figure 2F

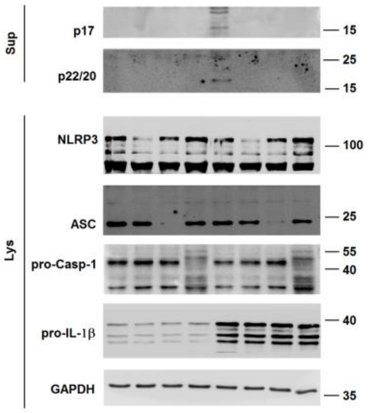

Uncropped Figure 2H

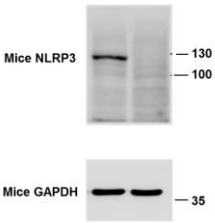

Uncropped Figure 2N

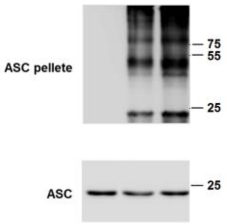

Uncropped Figure 3G

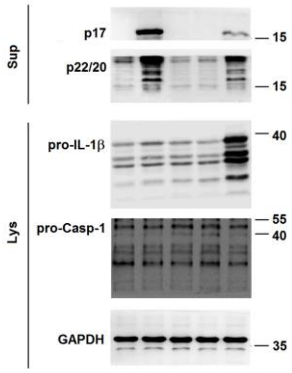

Uncropped Figure 3H

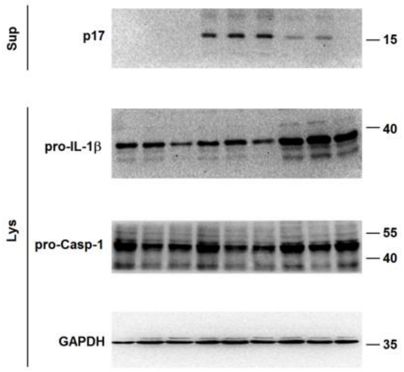

Uncropped Figure 3I

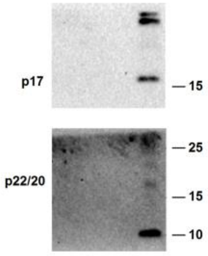

Uncropped Figure 4B

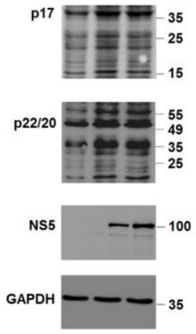

Uncropped Figure 4C

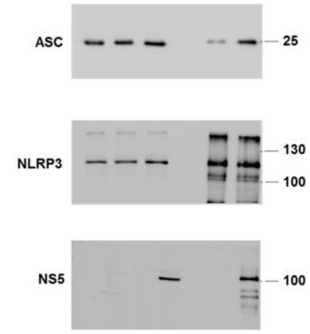

Uncropped Figure 4D

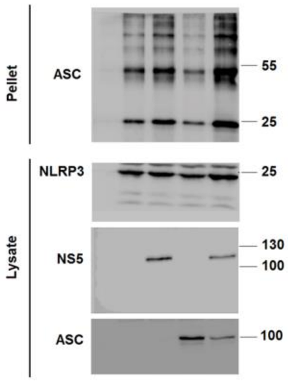

Uncropped Figure 4F

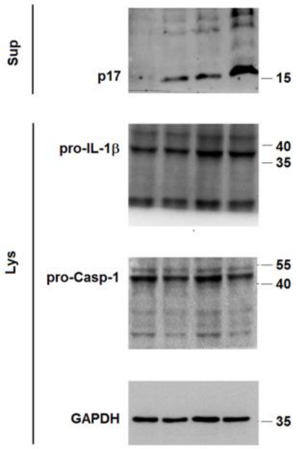

Uncropped Figure 4G

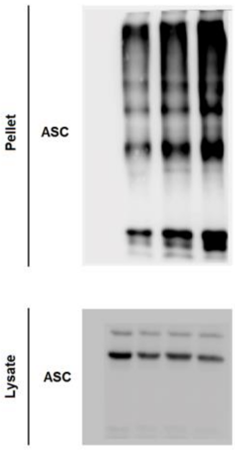

Uncropped Figure 4I

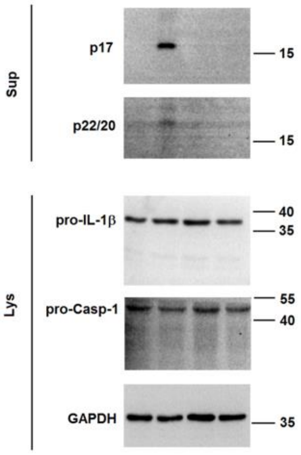

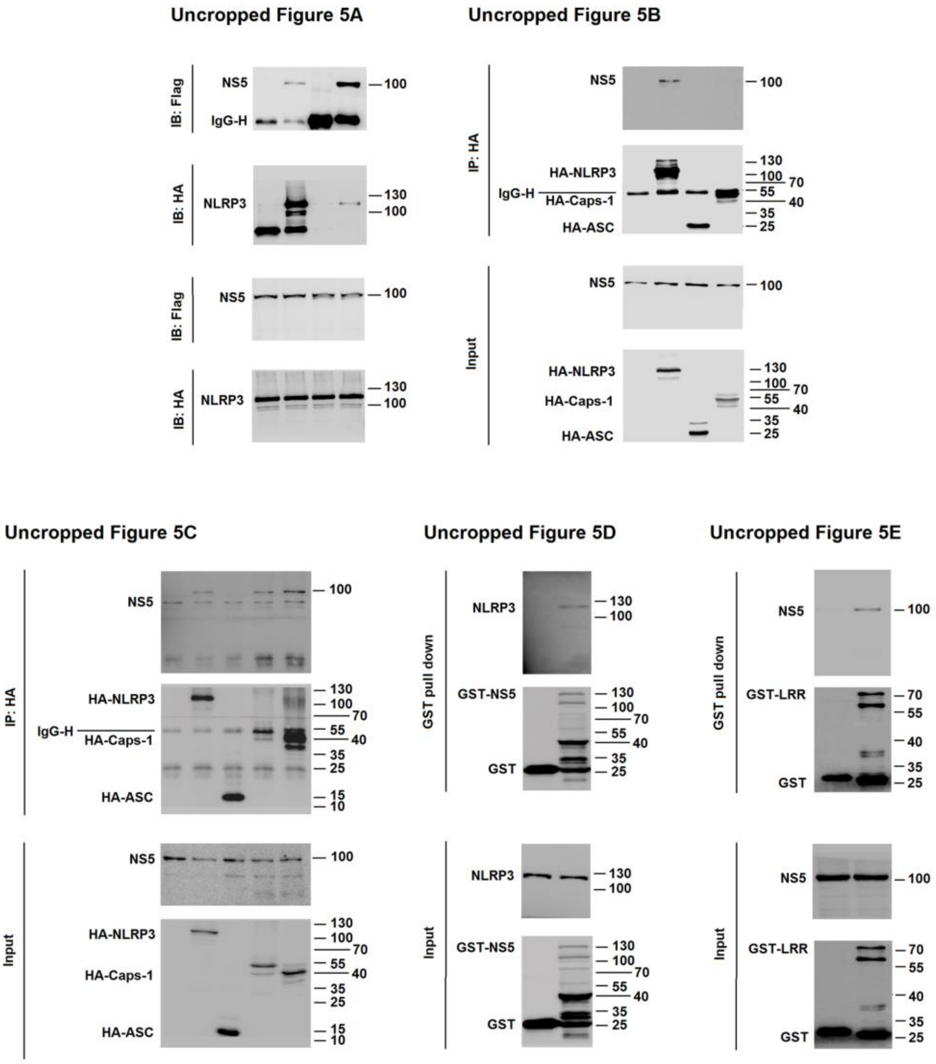

Supplementary Fig. 8. Uncropped scans of western blots.

Supplementary Tables

Supplementary Table 1. Primers used in this study for the construction of plasmids.

**Plasmids and genes**	**Sense primers**	**Anti-sense primers**
pcDNA3.1(+)-*NLRP3*	5’-CGCGGATCCATGAAGATGGCAAGCACCCGC-3’	5’-CCGCTCGAGCTACCAAGAAGGCTCAAAGAC-3’
pcDNA3.1(+)-*ASC*	5’-CCGGAATTCATGGGGCGCGCGCGCGACGCCAT-3’	5’-CCGCTCGAGTCAGCTCCGCTCCAGGTCCTCCA-3’
pcDNA3.1(+)-*Casp-1*	5’-CGCGGATCCATGGCCGACAAGGTCCTGAAG-3’	5’-CCGCTCGAGTTAATGTCCTGGGAAGAGGTA-3’
pcDNA3.1(+)-*IL-1bata*	5’-CCGGAATTCATGGCAGAAGTACCTGAGCTC-3’	5’-CCGCTCGAGTTAGGAAGACACAAATTGCAT-3’
pcagg-HA-*NS5*	5’-CCGGAATTCATGGGGGGTGGAACAGGAGAG-3’	5’-CCGCTCGAGCAGCACTCCAGGTGTAGACCC-3’
pcagg-HA-*NLRP3*	5’-TACGAGCTCATGAAGATGGCAAGCACCCGC-3’	5’-CCGCTCGAGCCAAGAAGGCTCAAAGACGAC-3’
pcagg-HA-*PYRIN*	5’-CCGGAATTCATGAAGATGGCAAGCACCCGC-3’	5’-CCGCTCGAGTAAACCCATCCACTCCTCTTC-3’
pcagg-HA-NACHT	5’-CCGGAATTCATGCTGGAGTACCTTTCGAGA-3’	5’-CCGCTCGAGGATCTTGCAACTTAATTTCTT-3’
pcagg-HA-*LRR*	5’-ATCGAGCTCATGTCTCAGCAAATCAGGCTG-3’	5’-CCGCTCGAGCCAAGAAGGCTCAAAGACGAC-3’
pcagg-HA-*ASC*	5’-CCGGAATTCATGGGGCGCGCGCGCGACGCC-3’	5’-CCGCTCGAGGCTCCGCTCCAGGTCCTCCAC-3’
pcagg-HA-*Caspase-1*	5’-CCGGAATTCATGGCCGACAAGGTCCTGAAG-3’	5’-CCGCTCGAGATGTCCTGGGAAGAGGTAGAA-3’
pGEX-6p-1-*NS5*	5’-CCGGAATTCATGGGGGGTGGAACAGGAGAG-3’	5’-CCGCTCGAGTTACAGCACTCCAGGTGTAGA-3’
pGEX-6p-1-*LRR*	5’-CGCGGATCCATGTCTCAGCAAATCAGGCTG-3’	5’-CCGCTCGAGCTACCAAGAAGGCTCAAAGAC-3’
pcDNA3.1(+)-3×Flag-*NS2A*	5’-GCGGATCCGGATCAACTGATCACATGGA-3’	5’-GCGAATTCCCGCTTCCCACTCCTTGTGA-3’
pcDNA3.1(+)-3×Flag-*NS2B*	5’-GCGGATCCAGCTGGCCCCCTAGCGAAGT-3’	5’-GCGAATTCCCTTTTTCCAGTCTTCACGT-3’
pcDNA3.1(+)-3×Flag-*NS4B*	5’-CGGGATCCAATGAACTCGGATGGTTGGA-3’	5’-GCGAATTCACGTCTCTTGACCAAGCCAG-3’
pcDNA3.1(+)-3×Flag-*NS5*	5’-CCGGAATTCTATGGGGGGTGGAACAGGAGA-3’	5’-CCGCTCGAGTTACAGCACTCCAGGTGTAGA-3’
pLenti-*NS5*	5’-ATTGATATCATGGGGGGTGGAACAGGAGAG-3’	5’-TTCCGCGGCCGCTATGGCCGACGTCGACTTACAGCACTCCAGGTG-3’

pGBKT7-*NLRP3*	5’-GGGAATTCCATATGATGAAGATGGCAAGCA-3’	5’-CGCGGATCCCTACCAAGAAGGCTCAAAGAC-3’
pGBKT7-*PYRIN*	5’-CCGGAATTCATGAAGATGGCAAGCACCCGC-3’	5’-CGCGGATCCCTATAAACCCATCCACTCCTC-3’
pGBKT7-*NACHT*	5’-CCGGAATTCATGCTGGAGTACCTTTCGAGA-3’	5’-CGCGGATCCCTAGATCTTGCAACTTAATTT-3’
pGBKT7-*LRR*	5’-CGCGGATCCATATGTCTCAGCAAATCAGGC-3’	5’-CCGCTCGAGCTACCAAGAAGGCTCAAAGAC-3’
pGADT7-*NS5*	5’-CCGGAATTCATGGGGGGTGGAACAGGAGAG-3’	5’-CCGCTCGAGTTACAGCACTCCAGGTGTAGA-3’

Supplementary Table 2. Primers used in this study for real-time PCR

**Primers**	**Sequences**
ZIKV forward	5'-GGTCAGCGTCCTCTCTAATAAACG-3'
ZIKV reverse	5'-GCACCCTAGTGTCCACTTTTTCC-3'
NLRP3 forward	5'-AAGGGCCATGGACTATTTCC-3'
NLRP3 reverse	5'-GACTCCACCCGATGACAGTT-3'
ASC forward	5'-AACCCAAGCAAGATGCGGAAG-3'
ASC reverse	5'-TTAGGGCCTGGAGGAGCAAG-3'
Casp-1 forward	5'-TCCAATAATGCAAGTCAAGCC-3'
Casp-1 reverse	5'-GCTGTACCCCAGATTTTGTAGCA-3'
IL-1β forward	5'-CACGATGCACCTGTACGATCA-3'
IL-1β reverse	5'-GTTGCTCCATATCCTGTCCCT-3'
TNF-α forward	5'-GGGTTTGCTACAACATGG-3'
TNF-α reverse	5'-AAGAACTTAGATGTCAGTGC-3'
IL-8 forward	5'-ACTTCTCCACAACCCTCTGC-3'
IL-8 reverse	5'-GTTGCTCCATATCCTGTCCCT-3'
GAPDH forward	5'-AAGGCTGTGGGCAAGG-3'
GAPDH reverse	5'-TGGAGGAGTGGGTGTCG-3'
